# Effects of Long-Term Oral Administration of *N*-Palmitoylethanolamine in Subjects with Mild Cognitive Impairment: Study Protocol

**DOI:** 10.3390/brainsci13081138

**Published:** 2023-07-29

**Authors:** Michela Bossa, Ornella Argento, Chiara Piacentini, Nicola Manocchio, Lucia Scipioni, Sergio Oddi, Mauro Maccarrone, Ugo Nocentini

**Affiliations:** 1 Behavioral Neuropsychology Laboratory, I.R.C.C.S. “Santa Lucia” Foundation, 00179 Rome, Italy; o.argento@hsantalucia.it (O.A.); c.piacentini@hsantalucia.it (C.P.); u.nocentini@hsantalucia.it (U.N.); 2 Department of Clinical Sciences and Translational Medicine, University of Rome “Tor Vergata”, 00133 Rome, Italy; nicola.manocchio@gmail.com; 3 Department of Biotechnological and Applied Clinical Sciences, University of L’Aquila, Via Vetoio Snc, 67100 L’Aquila, Italy; lucia.scipioni@graduate.univaq.it (L.S.); mauro.maccarrone@univaq.it (M.M.); 4 European Center for Brain Research/I.R.C.C.S. “Santa Lucia” Foundation, Via del Fosso di Fiorano 64, 00143 Rome, Italy; soddi@unite.it; 5 Faculty of Veterinary Medicine, University of Teramo, Via R. Balzarini 1, 64100 Teramo, Italy

**Keywords:** AD, cognition, MCI, neuroinflammation, PEA

## Abstract

*N*-palmitoylethanolamine (PEA) plays a key role in preventing Aβ-mediated neuroinflammation and neurotoxicity in murine models. It has been demonstrated that PEA provides anti-neuroinflammatory, pain-relieving and neuroprotective actions even in humans. In this project, we aim to evaluate these anti-neuroinflammatory effects via the cognitive evaluation and biochemical analyses of a 12-month oral administration of PEA in subjects with mild cognitive impairment (MCI). Subjects with MCI will be randomized to placebo or PEA groups, and followed for another 6 months. Cognitive abilities and neurological inflammation will be examined at baseline and after treatment. The specific objectives of the project are to ascertain whether: (i) PEA influences the scores of the neuropsychological and behavioral evaluations after one-year treatment, comparing PEA-treated and placebo subjects in both MCI and control groups; (ii) PEA can change the inflammatory and neuronal damage markers of blood and urine in MCI subjects; and (iii) these changes correlate with the clinical scores of participating subjects.

## 1. Introduction

Alzheimer’s disease (AD) is the most common type of dementia; its consequences dramatically affect patients and their families’ lives [1]. The progressive population aging on a global scale will lead to an increase in the number of people affected by AD, with high costs for national healthcare systems. Therefore, an early diagnosis could be crucial for the better management of AD, and therapeutic intervention at a preclinical stage of the disease may have a better chance of postponing the patient’s loss of autonomy, with obvious consequences in economic terms as well.

Despite decades of intensive research, AD remains incurable. Current therapies mainly focus on cholinergic and N-methyl-D-aspartate receptor pathways, providing only symptomatic relief, while several drugs targeting Aβ and tau have failed in Phase III clinical trials. Thus, the identification of novel therapeutic targets in order to prevent or delay disease progression appears urgent.

It is well known that mild cognitive impairment (MCI) represents a state of transition between normal cognition and dementia, particularly AD. It is estimated that its prevalence ranges between 15% and 20% after the age of 60, with an annual conversion rate to dementia up to 15% per year [2].

In this context, it could be particularly relevant to intervene in subjects with mild cognitive impairment (MCI) through nutraceutical, non-pharmacological treatments that could help arrest/slow down conversion to AD.

Among the nutritional substances that may be used to positively affect cognitive decline in elderly, *N*-palmitoylethanolamine (PEA) appears to come to the forefront owing to its high efficacy/risk ratio and lack of both tolerance induction and interference with other conventional therapies used for counteracting mental decline. PEA is an endogenous bioactive lipid biochemically and functionally related to endocannabinoids, though it does not bind to the same receptors [3]. Similarly to its congener, *N*-arachidonoylethanolamine (or anandamide, AEA), a major endocannabinoid [4], PEA acts as local mediator and its physiological tone depends on a finely regulated balance between biosynthesis, mainly catalyzed by NAPE-selective phospholipase D (NAPE- PLD), and degradation, mainly catalyzed by fatty acid amide hydrolase (FAAH) and *N*-acylethanolamine-hydrolyzing acid amidase (NAAA) [5,6]. Within the brain, PEA is produced “on demand” by neurons, microglia and astrocytes, thus having a pleiotropic, pro-homeostatic role in neurochemical and neuroimmune responses to various injurious processes, including those associated with AD [7,8]. In this context, growing preclinical evidence in animal models of β-amyloidosis shows that PEA is efficacious in preventing neuroinflammation, cognitive deficit and Aβ-mediated neurotoxicity, likely via peroxisome proliferator-activated receptor (PPAR)-α-dependent mechanism [9,10,11]. A growing body of preclinical studies attests the anti-neuroinflammatory, pain-relieving and neuroprotective actions of micronized/ultra-micronized formulations of PEA, which favor its oral bioavailability over unprocessed PEA [12,13].

PEA appears to have a good safety profile. Unlike other endocannabinoids, only relatively inactive products such as palmitic acid and ethanolamine are produced by PEA catabolism; these compounds do not seem to produce significant adverse effects [14]. Clinical protocols seem to confirm the safety of PEA application in real life too. In a recent meta-analysis [15] of 12 studies on the use of micronized and ultra-micronized PEA on chronic and/or neuropathic pain, the authors found PEA to be an effective treatment for pain and registered no serious adverse effects. Moreover, in the work of Bacci et al. [16] regarding the efficacy of PEA in reducing swelling and pain after dental surgery, only two in thirty patients reported adverse events: an episode of tachycardia, which developed about an hour after taking the medication; and an episode of drowsiness after taking the pill. These data seem to confirm that PEA use in in vivo clinical trials is an effective and safe option.

At the same time, an AD-like animal model of Tg2576 mice, aged 10/12 months, had a reduced concentration of PEA and its congener, AEA, when compared to age-matched wild-type littermates. Notably, MCI subjects had reduced plasma levels of PEA compared with age-matched controls. In agreement with these data, the key PEA-hydrolyzing enzyme, FAAH, is significantly up-regulated in peripheral monocytes isolated from AD patients, and its level correlates with the severity of the disease, suggesting that PEA signaling is impaired during AD progression [17].

The main hypothesis of this study is that, as AD is a neurodegenerative disorder with a prominent inflammatory component, AD patients could benefit from the assumption of PEA. To date, the effectiveness of PEA as an adjuvant for the management of cognitive decline in subjects with MCI has not been explored. Therefore, we aim to explore its effectiveness in managing AD-related neuroinflammation and interfering with the progression of the disease through neurodegeneration.

## 2. Materials and Methods

### 2.1. Aims, Design and Setting of the Study

This study has the following specific aims:

- Aim 1: To determine if PEA has any effect on neuropsychological, behavioral and functional assessment scores after one-year treatment by comparing treated and placebo arms, both in MCI and control groups;
- Aim 2: To determine if PEA modifies inflammatory and neuronal damage markers, both at the blood and urine levels in MCI subjects;
- Aim 3: To determine if these changes correlate with clinical scores in all study participants.

This study will be a double-blind placebo-controlled randomized clinical trial, performed on MCI subjects according to Petersen [18] and the International Working Group for people with amnestic MCI criteria [19]. The initial screening of suitable participants with MCI will take place at outpatient-specific facilities of the “Santa Lucia” Neuroscience and Rehabilitation Foundation (Rome).

- Aim 1:

During the recruitment period (0–12 months), an evaluation of the demography, history of concomitant diseases, dietary habits, body mass index (BMI), Mini Mental State Examination (MMSE; [20,21]) and Clinical Dementia Rating scale (CDR; [22]) assessment will be performed for screening all participants, according to the cognitive assessment tools most widely used in Italy. Then, all suitable subjects will be enrolled in the project.

At baseline (T0), a full cognitive, behavioral and functional assessment will be performed by administration of the Repeatable Battery for the Assessment of Neuropsychological Status (RBANS; [23]), Hospital Anxiety and Depression Scale (HADS; [24]) and Instrumental Activities of Daily Living (IADL) questionnaires [25]. Then, a dual-task (DT) protocol will be assessed [26].

All subjects will be randomly assigned 1:1, with the use of a numeric sequence standard procedure, into treated-with-PEA (composed of 600 mg PEA in capsules; orally, two times per day) or placebo (capsules; orally, two times per day) arms. Both MCI subjects and controls will be divided into two arms: for the MCI group, 25 subjects will be randomized in the PEA arm and 25 subjects in the placebo arm; for control group, 15 controls will be randomized in the PEA arm and 15 in the placebo arm. At the time of enrollment, all participants will be assigned to one of the two main groups (MCI or HC), then they will be assigned a sequential number. Following the random generation sequencing program (www.randomizer.org), each participant will be automatically assigned to one of the two treatment conditions without the direct involvement of any researcher, in order to avoid any personal bias.

Treatment will last for 12 months (T1), followed by a 6-month observational period (T2). All evaluations performed at T0 will be reassessed at T1 and T2.

- Aims 2 and 3:

In addition to the above, blood and urine specimens will be obtained from all participants at T0, T1 and T2. All samples will be immediately stored and then analyzed for measuring PEA, its endocannabinoid congeners and other biological markers.

The planned duration of the study is 36 months considering subject enrollment, clinical evaluation, sample collection, treatment duration, post-treatment follow-up, processing, interpretation and result disclosure.

**Figure 1:**
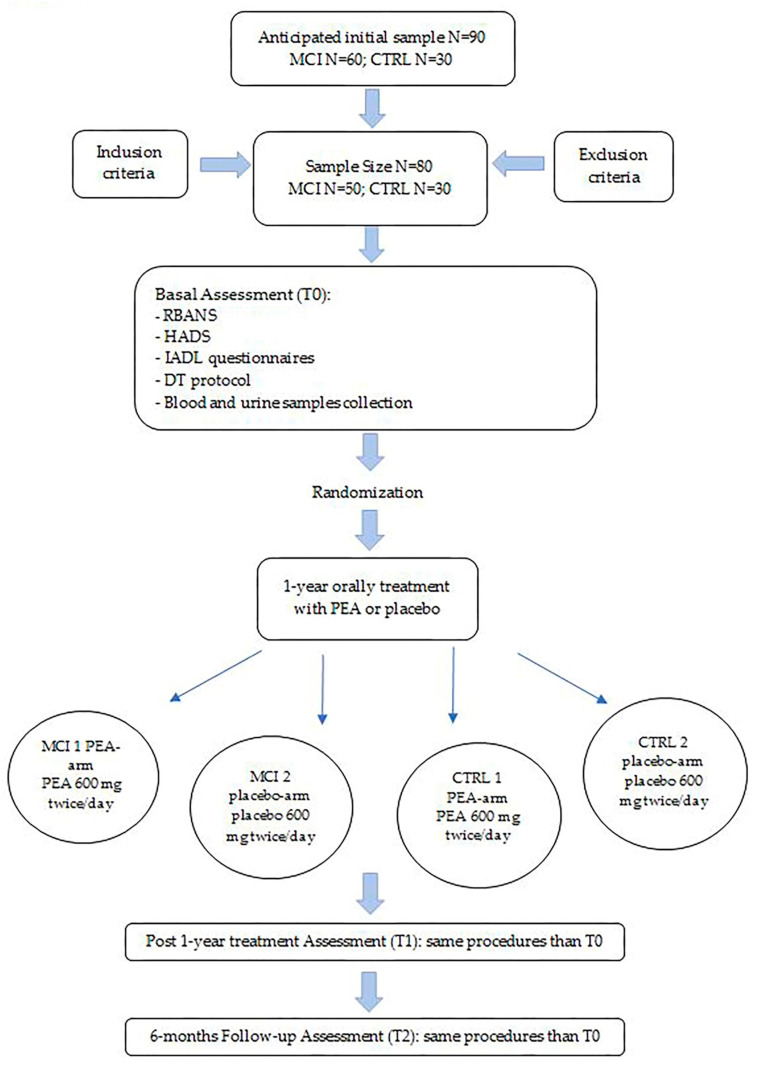
Study flowchart. CTRL: controls; DT: dual task; HADS: Hospital Anxiety and Depression Scale; IADL: Instrumental Activities of Daily Living; MCI: mild cognitive impairment; PEA: N-palmitoylethanolamine; RBANS: Repeatable Battery for the Assessment of Neuropsychological Status; T0: time 0; T1: time 1; T2: time 2.

Figure 1 shows the study flowchart.

### 2.2. Recruitment and Screening of Participants

Patients will be enrolled in the experimental group according to the following criteria:

Inclusion criteria: aged 60–89 years, possession of the capacity for making decisions, have a reference person (caregiver or companion), be able to read and write in Italian, a MMSE score between 23 and 26.9 points [20,21] and a CDR score < 0.5 [22].

Exclusion criteria: severe physical comorbidity that may limit the execution of the DT protocol, current significant neurological disease (e.g., diagnosis of Dementia, traumatic brain injury, etc.), a current history of alcoholism and/or substance abuse.

A parallel CTRL group of cognitively intact (MMSE score ≥ 27 points [20,21]) age- and sex-matched subjects will be also recruited. Screenings will be conducted by trained physicians and/or neuropsychologists.

We predicted an initial sample of 90 subjects, of which 60 are MCI and 30 CTRL. Then, we hypothesized that the sample size would be reduced due to the inclusion and exclusion criteria (i.e., a worsening in the cognitive functioning corresponding to an MMSE score < 23 points, and a reduced therapeutic adherence, more likely in the MCI group) and possible drop-outs. Therefore, the predicted final sample comprises 80 subjects, of which 50 are MCI and 30 CTRL.

All patients will be informed about the purposes of the research, and all procedures will be carried out with the appropriate understanding and written consent of the subjects, in accordance with the Declaration of Helsinki. Approval of the whole project will be obtained from the Independent Local Ethical Committee.

The inclusion and exclusion criteria are shown in Table 1.

### 2.3. Assessment Procedures

#### 2.3.1. Humans Cognitive, Behavioral and Functional Disability Assessment

- The RBANS battery [23] will be used for cognitive assessment. This neuropsychological battery measures cognitive decline, even in very mild dementia, or improvement across different domains: Immediate Memory, Visuospatial/Constructional Abilities, Language, Attention, Delayed Memory.
- HADS [24] will be used for behavioral assessment. It is useful in identifying the anxiety and depression levels of participants.
- IADL [25] is an appropriate instrument to assess independent living skills at a functional level.
- The DT [26] protocol assesses the cognitive–motor efficiency when simultaneously performing a cognitive and a motor task. It seems to be a reliable tool even in pre-dementia stages and consists of performing three 2 min tasks, two neutrals (motor task, cognitive task) and the other one with the concurrent tasks.

All these evaluations will be repeated after one-year PEA/placebo treatment and at the follow-up visit.

#### 2.3.2. Biochemical Analyses

Biological samples from subjects at T0, T1 and T2 will be aliquoted, stored at −80 °C until the evaluation of the following biochemical parameters:

- Apolipoprotein (apo) E genotyping: ApoE genotype of each participant will be determined via PCR, according to standard procedures. Humans have three versions of the APOE gene: ε2, ε3 and ε4 alleles. ApoE genotyping can help estimate a patient’s risk of converting from MCI to AD. In fact, in precedent studies, the epsilon4 allele of apolipoprotein E was considered as the major genetic risk factor for AD onset [27] and conversion to dementia in MCI patients [28].
- Central and peripheral inflammation: Selected inflammatory markers, specifically altered in MCI and possibly providing predictive information in AD progression, namely, C-reactive protein, interleukin (IL)-6, IL-8, IL-10, monocyte chemotactic protein-1, macrophage inflammatory protein-1beta and soluble triggering receptor expressed on myeloid cells 2, will be evaluated via ELISA and/or the Luminex platform.
- Amyloid-β, tau and neurofilament light chain: To monitor disease progression and treatment response, neurofilament light-chain protein, Aβ42, total and phosphorylated tau will be quantified via ELISA or using the multiplex Luminex platform.
- Oxidative stress: The effects of PEA treatment on oxidative/nitrosative stress that occurs at an early stage in AD will be assessed by measuring the presence of 3-nitrotyrosine and 4-hydroxynonenal (two end products of peroxynitrite), through ELISA [29].
- PEA, AEA and their main catabolic enzymes: The levels of PEA and AEA will be assessed via state-of-the-art liquid chromatography/mass spectrometry. In parallel, enzymatic activity of FAAH and NAAA [5] will be measured via radiometric assays.

## 3. Statistics and Outcomes

### 3.1. Data Analysis

The sample size needed to achieve significant differences was determined based on an a priori power analysis, using the software G*Power 3.1 (Version 3.1.9.4; Program written by Franz Faul, Universatat Kiel, Germany) [30] and published data on changes of Aβ42 levels in CSF of MCI vs. CTRL [31]. For a two-sample *t*-test, accepting a probability of type I error of 5% and a probability of type II error of 20%, required the following sample size for the outcome: Aβ 42 (ngl/mL), delta 42, SD 52, results to be N = 25/group. Based on the above, we propose to enroll 30 subjects per group, with the intent of having 23–25 finishers per group to account for potential dropouts. For the aims of our study, we plan to also enroll 30 control subjects that will be equally distributed within the PEA and placebo arms.

Experimental data will be analyzed using Student’s *t*-test or one- and two-way analyses of variance, followed by Bonferroni post hoc test for multiple comparisons. Differences between the groups will be considered statistically significant at values of *p* < 0.05. Pairwise correlation between variables will be assessed via Pearson’s test and confirmed using Spearman’s rank. Significant outliers will be excluded performing the Grubbs’ test, again, with statistically significant values considered at *p* < 0.05.

### 3.2. Outcomes

For the primary outcome of the study, we expect that PEA administration will decrease the levels of neuroinflammation markers and improve cognitive performance in MCI subjects.

Moreover, for the secondary outcome, we expect to find a reduced conversion from MCI to AD in subjects treated with prolonged PEA administration compared to those within the placebo arm.

For the last outcome, we will also identify the PEA-induced biochemical profile of selected AD targets and its correlation with MCI subjects’ cognitive performance, gaining new insight into biological mechanisms underlying PEA effects on amyloidosis-induced pathology.

## 4. Discussion and Conclusions

New drugs able to act on pathogenic mechanisms of AD may cover therapeutic areas where there are few or no treatments. To date, PEA is clinically used for conditions other than AD, and its safety and tolerability have been well-documented. Our proposal may have a significant impact on individual health and economic and social burdens. The possibility of a new therapeutic strategy in the early phases of the disease could provide therapies with the potential to alleviate the symptoms and also modify the disease progression. The quality of life in elderly and healthy aging populations has a remarkable societal impact. Today, approximately 50 million people worldwide live with dementia and this number is expected to increase to about 152 million by 2050; hence, AD may be considered an epidemic with an impact not only on patients and their families, but also on the worldwide healthcare system.

This project will open the gates to the use of substances, devoid of harmful effects, able to act on a specific mechanism that causes cellular damage typical of AD and, therefore, ways to delay the possible progression of MCI subjects to the AD stage.

## Figures and Tables

**Table 1:** Inclusion and exclusion criteria for the enrollment of patients.

Inclusion Criteria	
Diagnosis (for MCI group)	MMSE score 23–26.9 points; CDR scale score <0.5 points
Age	60–89 years
Cognitive status	Possess the capacity for making decisions
Language	Be able to read and write in Italian
Other conditions (for MCI group)	Have a reference person (namely, informant)
Exclusion criteria	
Pathologies	Current significant neurological disease (e.g., diagnosis of dementia, traumatic brain injury, etc.)
Motor limitations	Severe physical comorbidity
Medications	Cognitive enhancer medication
Other conditions	Current history of alcoholism and/or substance abuse

CDR: Clinical Dementia Rating; MCI: mild cognitive impairment; MMSE: Mini Mental State Examination.

## Data Availability

Not applicable.
